# Biology and Clinical Applicability of Plasma Thymus and Activation-Regulated Chemokine (TARC) in Classical Hodgkin Lymphoma

**DOI:** 10.3390/cancers13040884

**Published:** 2021-02-20

**Authors:** Eline A. M. Zijtregtop, Iris van der Strate, Auke Beishuizen, Christian M. Zwaan, Marijn A. Scheijde-Vermeulen, Arianne M. Brandsma, Friederike Meyer-Wentrup

**Affiliations:** 1 Department of Pediatric Hematology and Oncology, Erasmus Medical Center-Sophia Children’s Hospital, 3015 GD Rotterdam, The Netherlands; e.zijtregtop@erasmusmc.nl (E.A.M.Z.); A.Beishuizen-2@prinsesmaximacentrum.nl (A.B.); c.m.zwaan@prinsesmaximacentrum.nl (C.M.Z.); 2 Department of Pediatric Hemato-oncology, Princess Máxima Center for Pediatric Oncology, 3584 CS Utrecht, The Netherlands; iris.vd.strate@hotmail.com (I.v.d.S.); A.M.Brandsma@prinsesmaximacentrum.nl (A.M.B.); 3 Department of Pathology, Princess Máxima Center for Pediatric Oncology, 3584 CS Utrecht, The Netherlands; M.A.Vermeulen-16@prinsesmaximacentrum.nl

**Keywords:** thymus and activation-regulated chemokine (TARC), biomarker, classical Hodgkin lymphoma, lymphoma biomarker

## Abstract

**Simple Summary:**

Thymus and activation-regulated chemokine (TARC) is an important biomarker in classical Hodgkin lymphoma and several other diseases. The exact role of TARC in the pathogenesis of these diseases, as well as its therapeutic potential, is not yet fully understood. Understanding the role of TARC in Hodgkin lymphoma is important since it might help in risk stratification of patients and it could be a therapeutic target. We give an overview of the biological functions of TARC and its potential as biomarker and therapeutic target.

**Abstract:**

Thymus and activation-regulated chemokine (TARC) is produced by different cell types and is highly expressed in the thymus. It plays an important role in T cell development, trafficking and activation of mature T cells after binding to its receptor C-C chemokine receptor type 4 (CCR4) and consecutive signal transducer and activator of transcription 6 (STAT6) activation. Importantly, TARC is also produced by malignant Hodgkin and Reed–Sternberg (HRS) cells of classical Hodgkin lymphoma (cHL). In cHL, HRS cells survive and proliferate due to the micro-environment consisting primarily of type 2 T helper (Th2) cells. TARC-mediated signaling initiates a positive feedback loop that is crucial for the interaction between HRS and T cells. The clinical applicability of TARC is diverse. It is useful as diagnostic biomarker in both children and adults with cHL and in other Th2-driven diseases. In adult cHL patients, TARC is also a biomarker for treatment response and prognosis. Finally, blocking TARC signaling and thus inhibiting pathological Th2 cell recruitment could be a therapeutic strategy in cHL. In this review, we summarize the biological functions of TARC and focus on its role in cHL pathogenesis and as a biomarker for cHL and other diseases. We conclude by giving an outlook on putative therapeutic applications of antagonists and inhibitors of TARC-mediated signaling.

## 1. Introduction

Classical Hodgkin lymphoma (cHL) is a malignancy of the lymphatic system with an incidence of 2–3/100,000 per year in developed countries [1]. Generally, cHL occurs in all age groups. It has a unique bimodal age distribution with a peak in the adolescent/young adult (AYA) population (15 to 35 years) and another after the age of 55 years [2]. cHL accounts for 15% to 25% of all lymphomas and represents the most common lymphoma subtype in children and young adults in the Western world [3].

Nowadays, cHL is a highly curable malignancy in all age groups. The 5-year relative survival for patients aged from 0 to 19 years is 96.4%, and 89.8% for those diagnosed between ages 20 and 64 years [4]. Anthracycline-based chemotherapy with or without radiation is the mainstay of cHL treatment [5,6]. Advances in understanding the biology of the disease and improvement in modalities of chemotherapy and radiotherapy have improved survival in every stage of cHL [3]. However, patients with advanced-stage or high-risk disease are only cured in approximately 70% of cases and high-dose chemotherapy in combination with autologous stem-cell transplantation (ASCT) is only successful in half of the patients with relapsed/refractory cHL [7]. Moreover, especially in the AYA group, treatment-related toxicities among which second malignancies, cardiovascular and lung complications and fertility problems are of great concern [8,9,10,11]. Thus, the challenge remains to tailor treatment to eradicate malignancy with minimal side effects and to simultaneously identify those patients in whom alternative strategies should be initiated early.

cHL is a peculiar malignancy, because the malignant Hodgkin and Reed–Sternberg cells (HRS cells) are greatly outnumbered by immune cells in the tumor microenvironment. Only 0.1–10% of the tumor consists of HRS cells [12,13,14]. The microenvironment consists of T and B lymphocytes, eosinophils, macrophages, mast cells, plasma cells, and stromal cells. This lymphoma microenvironment supports growth and proliferation of HRS cells [15,16]. As a consequence, primary HRS cells do not grow in cell culture. Cell lines are rare and, in the absence of a microenvironment, only suitable for limited analysis of cell-intrinsic properties, as they do not reflect the physiological situation of the lymphoma in vivo [17]. These characteristics of cHL have impeded the development of preclinical models to study the disease. Progress in molecular techniques and new strategies, such as laser microdissection and fluorescence-activated cell sorting, has contributed to more insight into the pathogenesis, genetic alterations and immune escape mechanisms of cHL. However, the next challenge is to translate and implement this into the clinic [3].

As the impact of the microenvironment becomes increasingly clear, there is more focus on the implementation of therapeutic strategies targeting the tumor–host interactions [3]. Checkpoint inhibition, for example, has been implemented in current treatment protocols for adult patients with relapsed/refractory cHL [18,19]. More insight into the biology of the microenvironment will probably lead to additional improvements in treatment outcomes. In light of this success, many studies have focused on associations of the tumor microenvironment and blood biomarkers with patient outcomes. Blood biomarkers result from active crosstalk between HRS cells and the microenvironment [13,16]. The underlying biology of these relationships is only partly understood. Blood biomarkers potentially reflect lymphoma viability and therefore they may be very important for diagnosis, assessing disease extent and treatment stratification. Thymus and activation-regulated chemokine (TARC) has been the first and, so far, most potential blood biomarker characterized in cHL patients. Here, we will review the role of TARC in cHL, its relevance as a marker of disease activity and its potential as a therapeutic target.

## 2. Biology of TARC

Chemokines are a superfamily of potent leukocyte chemotactic cytokines found in many different species, including mammals, birds, and fish [20]. Chemokines are small, secreted, highly basic proteins that regulate cell trafficking and homing of leukocytes. They are expressed in tissues during normal homeostasis, but best known for their upregulation after injury or infection, to attract immune cells to the site of damage or inflammation. Indeed, most chemokines are produced under pathological conditions by both tissue cells and infiltrating leukocytes [21]. Chemokines and chemokine receptors were originally studied because of their role in inflammation but are now known to play a crucial part in directing migration and localization of immune cells in the body as well, enabling adaptive immune responses and contributing to the pathogenesis of various diseases [22].

TARC, also called C-C motif chemokine ligand 17 (CCL17), is an 8 kDa chemokine belonging to the CC chemokine family, which consists of chemokines with two adjacent conserved cysteine residues [20]. CC chemokines are chemotactic for many different cells of the immune system, including monocytes, eosinophils, basophils, lymphocytes, dendritic cells (DCs), and natural killer (NK) cells. TARC was the first CC chemokine identified to be chemotactic for lymphoid cells, but not for monocytes and granulocytes. Furthermore, it was the first CC chemokine not to be mapped to the major CC chemokine cluster on chromosome 17; TARC is mapped to chromosome 16q13 [23,24]. TARC is produced by DCs, endothelial cells, keratinocytes and fibroblasts, and highly expressed in the thymus, where it plays an important role in T cell development, trafficking and activation of mature T cells [23]. TARC binds to the G-protein coupled receptor CCR4 [23]. CCR4 is predominantly expressed on human T cells polarized to the type 2 helper (Th2) phenotype, although expression can also be found on other immune cells such as eosinophils, basophils, regulatory T cells, NK cells, systemic memory T cells, cutaneous lymphocyte-associate antigen (CLA)+ T cells and platelets. After stimulation, Th2 cells produce cytokines such as IL-4, IL-5, and IL-13 and activate B cells to induce humoral immunity. In addition, Th2 cells are known to play a central role in allergic diseases.

T cells can also produce TARC as human T cells stimulated with IL-4, the main Th2 cytokine, upregulate TARC expression [25]. IL-4 binds to the IL-4 receptor on T cells, leading to downstream Janus kinase (JAK)/signal transducer and activator of transcription (STAT) signaling. After phosphorylation and dimerization, STAT6 transfers to the nucleus to induce TARC transcription [25]. TARC expression in keratinocytes is co-localized with interferon-gamma (IFNγ) and tumor necrosis factor-alpha (TNFα), both pleotropic cytokines that play various roles in inflammatory and immune reactions [26]. Together, these two pro-inflammatory cytokines stimulate TARC production and secretion in keratinocytes, while transforming growth factor-beta (TGFβ) has the opposite effect [27]. In another study, it was found that TARC expression in murine Langerhans cells (LCs) was upregulated by IL-4 and TNFα but downregulated by IFNγ [28]. IFNγ binding to its receptor induces JAK/STAT signaling mainly through activation of STAT1, while TNFα can activate multiple signaling pathways including activation of the mitogen-activated protein kinase (MAPK) and NF-kappa B (NFκB) pathways. STAT1 was shown to be dispensable for IFNγ/TNFα-induced TARC production in keratinocytes [29]. Inhibiting NFκB or p38 mitogen-activated protein kinase (MAPK) activation did block the TARC production induced by IFNγ/TNFα [29], indicating that these cytokines use the NFκB/p38 MAPK signaling pathway for TARC induction in keratinocytes. Taken together, this indicates that the regulation of TARC production differs per cell type and most likely per location in the body.

Activated endothelium expresses TARC to regulate interactions with circulating lymphocytes. Endothelial cells in inflamed tissues express TARC to induce integrin-dependent adhesion of skin memory T cells through ICAM-1 causing their rapid arrest [30]. In this setting, only skin-homing memory T cells are attracted by TARC, whereas intestinal memory T cells poorly respond to TARC on inflamed endothelium [30]. This suggests an important role for TARC and CCR4 in homing tissue-specific T cells to their respective target tissues. In addition, TARC plays a role during platelet aggregation as CCR4 is expressed on platelets. TARC can induce concentration-dependent platelet aggregation, with low levels of platelet agonists, such as ADP or thrombin, required to initiate this process [20,31]. Platelets themselves also contain TARC, which is released after stimulation with thrombin, most likely attracting other immune cells to the site of clotting [32]. Importantly, platelets express many chemokine receptors and respond to many different chemokines, allowing them to respond to various signals and linking platelet activation to recruitment of specific immune cells depending on the location and signals present.

TARC and CCR4 signaling have been shown to play a role in different types of diseases, including atopic dermatitis (AD), allergic asthma and other (allergic) immune-mediated diseases [33,34,35]. Even though TARC-induced attraction of Th2-polarized T cells to sites of inflammation is considered to be an important factor, the exact mechanisms of how TARC contributes to pathogenesis or initiation of these diseases remain unknown.

## 3. TARC in Classical Hodgkin Lymphoma

**Figure 1:**
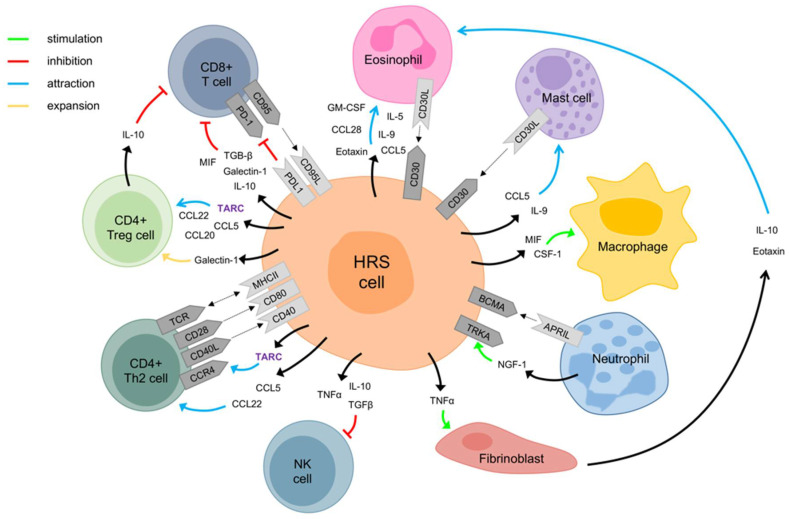
The figure shows interactions between Hodgkin and Reed–Sternberg (HRS) cells and the tumor microenvironment (direct cellular interactions and via soluble mediators). Many different cell types are attracted into the microenvironment by chemo- and cytokines. HRS cells attract CD4+ type 2 T helper (Th2) cells through secretion of the chemokines TARC, CC chemokine 5 (CCL5) and CCL22. These, as well as CCL20, also attract CD4+ regulatory T (Treg) cells. HRS cells are stimulated by neutrophils through APRIL-BCMA interaction and secretion of nerve growth factor-1 (NGF-1). NGF-1 binds to the receptor tyrosine kinase (TRKA) on HRS cells. HRS cells are also stimulated by mast cells and eosinophils by CD30-CD30 ligand interaction. CD8+ T cells, also known as cytotoxic T cells, are inhibited by IL-10, produced by Treg cells. HRS cells also inhibit CD8+ T cells and NK cells through IL-10 and other immunosuppressive mediators and expression of the programmed cell death 1 ligand (PD1L).

Two years after discovery of TARC, it was shown that yet another cell type can produce TARC: HRS cells, the malignant cells of cHL [36]. HRS cells and the lymphoma microenvironment communicate through expression of various different chemo- and cytokines (Figure 1) [12,13,14]. In cHL, TARC is produced by HRS and antigen-presenting cells [23], and possibly also by some IL-4 stimulated T cells (see above). In addition, HRS cells express CCR4 on their surface, making them susceptible to auto- and paracrine TARC stimulation. While the function of TARC in cHL has not been fully elucidated, the available data will be discussed below.

**Figure 2:**
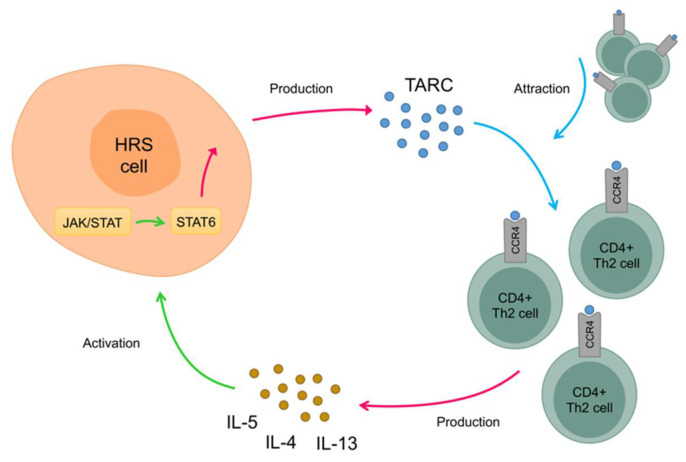
The interaction between Hodgkin and Reed–Sternberg (HRS) cells and CD4+ type 2 T helper (Th2) cells most likely initiates a positive feedback loop. First, the secretion of TARC (blue circles) by HRS cells causes a consequent attraction and homing of Th2 cells to the tumor microenvironment. These attracted Th2 cells can secrete IL-4, IL-5 and IL-13 (yellow circles) that activate JAK/STAT signaling leading to activation of STAT6. STAT6 activation in HRS cells further increases TARC secretion. This leads to a feedback loop of constant stimulation of HRS cells.

The cHL microenvironment is essential in supporting HRS cells. The HRS cells reside among an overwhelming number of Th2 cells and secrete high levels of cytokines and chemokines, including several interleukins and TARC [12,15,16,25,37]. These cytokines bind to their surface receptors both on HRS cells and cells of the direct microenvironment, initiating JAK/STAT downstream signaling [37]. The interaction between HRS cells and Th2 cells most likely initiates a positive feedback loop. First, the secretion of TARC by HRS cells causes a consequent attraction and homing of Th2 cells to the tumor microenvironment [25,37]. These attracted Th2 cells can secrete IL-4, IL-5 and IL-13 that activate JAK/STAT signaling leading to activation of STAT6 [25]. STAT6 activation in HRS cells further increases TARC secretion [37]. Together, this leads to a feedback loop of constant stimulation of HRS cells, also shown in Figure 2 [25,37]. This stimulation might be further enhanced by genetic alterations that increase JAK/STAT signaling, with almost 90% of cHL containing genetic lesions involving one of the JAK/STAT pathway members [38]. These include lesions predicted to activate the pathway directly (JAK1, JAK2, STAT3, STAT5B, STAT6) or to inactivate inhibitory proteins such as SOCS1 or PTPN1 [38]. Activated STAT3 and STAT6 are frequently observed in cultured and primary HRS cells and are reported to promote their survival [37]. Treatment of cHL cell lines or cHL lymph node single cell suspensions with a JAK2 inhibitor reduced the cell viability to some extent [39]. In addition, blocking the JAK/STAT signaling by inhibiting heat-shock protein 90 (HSP90) reduced cell proliferation of cHL cell lines [40]. Together, these data indicate that HRS cells require continuous JAK/STAT signaling that can be obtained by TARC-mediated attraction of Th2 cells in combination with acquisition of genetic lesions resulting in further activation of the pathway.

A better understanding of cHL biology, and more specifically the role of TARC in survival of HRS cells, may lead to improved, more targeted treatment options. However, cHL pathogenesis and, in particular, the underlying genetic lesions have proven difficult to elucidate because of the scarcity of HRS cells and their dependence on the benign microenvironment. As a consequence, primary HRS cells are very difficult to culture in vitro. Cell lines are rare and the ones that exist grow in the absence of the microenvironment and are therefore not suitable for studying the molecular basis of the HRS cell–microenvironment interactions [17]. Animal models to study the HRS-microenvironment dependency do not exist. Analysis of patient-derived tumor tissue is therefore crucial for understanding the pathophysiology of cHL and more sophisticated in vitro models are needed. Progress in understanding the biology of cHL has required the laborious purification of HRS cells from tissues by laser microdissection or fluorescence-activated cell sorter sorting [41,42,43]. Data of comprehensive characterization of cHL by (single cell) RNA sequencing, whole-exome sequencing (WES) and whole-genome sequencing (WGS) are scarce but will hopefully expand in the future [44,45,46,47,48].

Since HRS cells produce high amounts of TARC that can be detected in lymphoma biopsies using immunohistochemistry and in serum of cHL patients, studies have focused on the use of TARC as a disease biomarker. This will be discussed in more detail below.

## 4. Clinical Applicability of TARC: As Diagnostic Blood Biomarker

**Figure 3:**
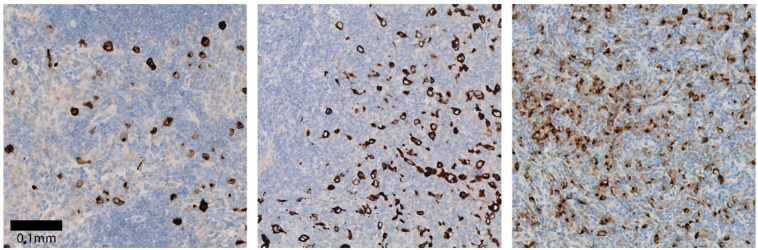
Positive TARC staining of HRS cells (brown) by immunohistochemistry in lymphoma tissues slides of three different pediatric cHL patients. Scale bar depicts 0.1 mm size, all images were made with the same magnification.

Lymph node biopsy is the gold standard for diagnosing cHL. This is a relatively time-consuming and expensive procedure, often requiring general anesthesia. Moreover, albeit small, there is a risk of complications. In patients with a large mediastinal mass, lymph node biopsy may be contraindicated due to danger of causing respiratory failure. For these reasons, diagnostic blood biomarkers that could confirm or exclude cHL diagnosis would add great value because they are more easily available and reproducible, cost-effective and almost non-invasive for the patient. TARC is known to be an important diagnostic biomarker in cHL patients. Approximately 90% of adult cHL biopsies show TARC-positive HRS cells by immunohistochemistry (Figure 3) and about 82–93% of patients have significantly elevated TARC levels in their pre-treatment serum [49,50,51,52]. We recently demonstrated that TARC is a highly sensitive and specific diagnostic biomarker for pediatric cHL as well [53]. In our series, 97.8% of the pediatric patients with cHL had elevated TARC levels (Figure 3). In plasma, a TARC cut-off level of 942 pg/mL gave a sensitivity of 97.9% (95% CI 88.7–100%) and specificity of 100% (95% CI 95.5–100%) [53]. Staging and response to treatment measurements in pediatric cHL patients require Fluor-Deoxyglucose-Positron Emission Tomography (FDG-PET) and Computed Tomography (CT) scans. Although these are sensitive tests, they have several disadvantages, such as exposure to radiation, time consumption, high costs and lack of specificity [54]. For staging and treatment evaluation, blood biomarkers could potentially have great added value on top of the standard procedures, especially if they have a higher sensitivity or specificity for disease activity than FDG-PET scans.

In adult cHL patients higher TARC levels correlate with the extent of the disease; higher TARC levels are correlated with higher disease stage, presence of B-symptoms, bulky disease and metabolic tumor volume [52]. TARC levels in children are associated with age, treatment level, bulky disease, B-symptoms and ESR [53]. In adult cHL patients, TARC is also correlated with treatment response [50,52,55,56,57]. High post-therapy levels of TARC are associated with poorer survival [58]. Early reduction in TARC after one cycle of chemotherapy was also associated with progression free survival (PFS) [50]. Recently, it was shown that the positive predictive value (PPV) of interim TARC levels is higher than the PPV of interim PET scans [59]. Interim TARC levels are associated with treatment failure, even within the subgroup of PET negative patients [60]. Elevated posttherapy TARC levels were correlated with shorter PFS and overall survival (OS) as well, even after adjusting for the International Prognostic Score and end of treatment PET status [61]. Research into the correlation between TARC levels and treatment response in children with cHL is ongoing.

TARC is used as biomarker in other diseases as well, making the potential applicability diverse. For most lymphomas other than cHL, TARC has no potential as a biomarker [62]. Possible exceptions are cutaneous T cell lymphomas and nasal natural killer/T cell lymphoma (NNKTL). In one study with NNKTL patients, TARC was highly upregulated in NNKTL cell lines and patient sera [63]. In a study with cutaneous T cell lymphoma patients, TARC was useful in making the diagnosis [64]. In anaplastic large cell lymphomas (ALCL), TARC is expressed in around half of the anaplastic lymphoma kinase (ALK)-negative ALCL and in none of the ALK-positive ALCL [65]. Thus, TARC is a potential biomarker in some T cell lymphomas.

The role of TARC in recruiting Th2 lymphocytes in diseases other than cHL support TARC playing a similar role in the pathogenesis of cHL [26,66]. We provide an overview of the use of TARC as biomarker for oncological (other than cHL) (Table 1) and non-oncological (Table 2) diseases.

## 5. Clinical Applicability of TARC: As Therapeutic Target

Although the overall survival of cHL is excellent, there is still a group of patients who suffer from refractory or relapsed disease [7,84,85]. Standard chemo- or radiotherapy regimens are not sufficient for these patients. In addition, standard treatment-related toxicities are of major concern, especially in children and young adults [8,9,10,11]. Therefore, novel therapeutic strategies are indispensable. Blocking TARC and stopping pathological Th2 recruitment into the afflicted lymph nodes may disrupt the microenvironment niche required by HRS cells for survival. This could induce HRS cell death and could therefore be beneficial for patients suffering from cHL. Chemokine signaling is successfully inhibited either by adding receptor antagonists, such as antibodies or soluble receptors that prevent chemokine-receptor (TARC-CCR4) binding, or by inhibiting downstream signaling molecules, such as STAT6. Most pharmaceutical approaches used in the clinic are directed at inhibiting the chemokine-receptor binding [84]. Anti-CCR4 antibody treatment can prevent binding of TARC to CCR4. This inhibits the recruitment of Th2 cells and could lead to a reduction in the supportive tumor microenvironment and positive feedback loop. In addition, regulatory T cells also respond to TARC via CCR4, indicating that blocking this interaction might reduce the regulatory T cell activity and thereby increase the potential of cytotoxic T cells to eliminate HRS cells.

Mogamulizumab (KW-0761) is a defucosylated, humanized anti-CCR4 immunoglobulin G1 (IgG1) monoclonal antibody that blocks the receptor. Reducing fucose content in the structure of the Fc region of the antibody enhances the antibody-dependent cellular cytotoxicity (ADCC) activity [85]. When mogamulizumab binds to CCR4, it induces ADCC against CCR4+ T cells. By this mechanism it kills T cells and NK cells [86,87]. Mogamulizumab is currently used for treating relapsed or refractory adult T cell leukemia-lymphoma in Japan [88]. Since mogamulizumab binds to CCR4, it may also directly interfere with the CCR4/TARC interaction on Th2 and regulatory T cells. As Th2 cells support the proliferation and survival of HRS cells, blocking the CCR4/TARC interaction or even directly eliminating these Th2 cells might lead to HRS cell apoptosis. In addition, regulatory T cells in the microenvironment also respond to TARC via CCR4 and inhibit cytotoxic CD8+ T cells from killing HRS cells. Mogamulizumab might reduce regulatory T cell activity in the microenvironment, thereby increasing the potential of cytotoxic T cells to eliminate HRS cells. Therefore, mogamulizumab may be a relatively specific and potent agent in the treatment of patients with cHL.

Vorinostat is an inhibitor of histone deacetylase (HDAC) activity [89]. It works at the epigenetic level to influence gene expression and can block cancer cell proliferation in vitro and in vivo [90]. Although vorinostat is not a specific inhibitor of TARC, it was shown to inhibit STAT6-mediated cytokine- and TARC production in Th2 cells, induce cell death in cHL cell lines, and induce p21 expression causing cell-cycle arrest and apoptosis in cHL cell lines [37]. STAT6 inhibition increases the rate of apoptosis and decreases the secretion of TARC in HRS cells [37]. Vorinostat is currently investigated in phase II trials for patients with relapsed or refractory cHL [91,92].

Another potential novel treatment for cHL is adoptive T cell therapy with CD30-specific chimeric antigen receptor (CAR) expression, especially when combined with enhanced migration to the tumor microenvironment. Effector CD8+ T cells normally lack CCR4 expression, but after retroviral transduction this could be induced together with CD30-CAR expression [93]. These genetically modified T cells showed enhanced migration towards tumor cells and could specifically kill CD30+ lymphoma cells, both in vitro and in vivo in cHL mouse models [93].

These examples are possible approaches for therapeutic TARC inhibition or exploitation of the TARC/CCR4 interaction in cHL. In addition, they underline the need for new in vitro and in vivo models to study the cHL-microenvironment interactions and to test the therapeutic potential of TARC inhibition in cHL. 

## 6. Conclusions

TARC is a chemokine that is involved in different cellular pathways. It is considered to play an important role in the pathogenesis of cHL and other malignant and non-malignant diseases. The clinical applicability of TARC in cHL ranges from its use as a diagnostic and prognostic biomarker to being a putative therapeutic target. Further research is required to investigate the precise role of TARC in the pathogenesis of cHL and the potential applicability of TARC inhibition to improve the outcome of cHL patients.

## Figures and Tables

**Table 1:** Potential applicability of TARC in oncological diseases.

Disease	Applicability	Ref ^1^
Gastric cancer	Diagnostic marker, marker of disease extent	[67]
Melanoma	Marker for progression free survival	[68]
Nasal natural killer/T cell lymphoma	Diagnostic marker	[63]
Anaplastic large cell lymphoma	Diagnostic marker in ALK-negative cases	[65]
Cutaneous T cell lymphoma	Diagnostic marker	[64]

^1^ Ref: Reference.

**Table 2:** Potential applicability of TARC in non-oncological diseases.

Disease	Applicability	Ref ^1^
Atopic dermatitis	Diagnostic marker, response marker, severity marker	[34,69]
Asthma	Diagnostic marker, severity marker, marker for exacerbation	[33,70]
Drug eruption	Severity marker	[71]
Chronic rhinitis	Characterize disease phenotype	[72]
IgG4-related disease	Role in pathogenesis	[73]
Alopecia areata	Disease activity and response marker	[74]
Generalized pustular psoriasis	Therapeutic response marker	[75]
Chronic obstructive pulmonary disease	Marker for decrease lung function	[76,77]
Ankylosing spondylitis	Role in pathogenesis	[78]
Acute eosinophilic pneumonia	Diagnostic marker	[79]
Bullous pemphigoid	Role in pathogenesis	[80]
Food protein-induced enterocolitis syndrome	Diagnostic marker	[81]
Bronchopulmonary Aspergillosis in cystic fibrosis	Diagnostic marker	[82]
Cutaneous lupus erythematosus	Role in pathogenesis	[83]
Systemic lupus erythematosus	Role in pathogenesis	[35]

^1^ Ref: Reference.

## Data Availability

No new data were created or analyzed in this study. Data sharing is not applicable to this article.
